# Cyclopiazonic Acid Biosynthesis of *Aspergillus flavus* and *Aspergillus oryzae*

**DOI:** 10.3390/toxins1020074

**Published:** 2009-11-06

**Authors:** Perng-Kuang Chang, Kenneth C. Ehrlich, Isao Fujii

**Affiliations:** 1 Southern Regional Research Center, Agricultural Research Service, US Department of Agriculture, 1100 Robert E. Lee Boulevard, New Orleans, LA 70124, USA; Email: ken.ehrlich@ars.usda.gov (K.E.); 2 School of Pharmacy, Iwate Medical University, 2-1-1 Nishitokuta, Yahaba, Iwate 028-3694, Japan; Email: ifujii@iwate-med.ac.jp (I.F.)

**Keywords:** *Aspergillus*, cyclopiazonic acid, gene cluster, non-ribosomal peptide synthase

## Abstract

Cyclopiazonic acid (CPA) is an indole-tetramic acid neurotoxin produced by some of the same strains of *A. flavus* that produce aflatoxins and by some *Aspergillus oryzae* strains. Despite its discovery 40 years ago, few reviews of its toxicity and biosynthesis have been reported. This review examines what is currently known about the toxicity of CPA to animals and humans, both by itself or in combination with other mycotoxins. The review also discusses CPA biosynthesis and the genetic diversity of CPA production in *A. flavus/oryzae* populations.

## 1. Introduction

**Figure 1:**
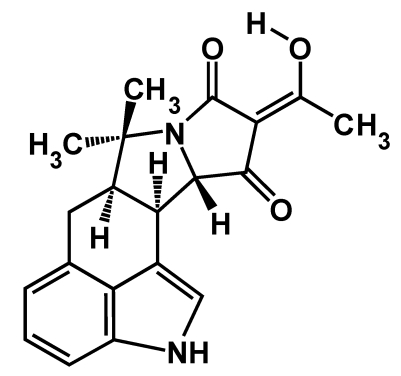
Structure of α-CPA.

Mycotoxins are fungal secondary metabolites which, if ingested, can evoke a wide range of toxic responses and disease conditions in higher vertebrates. Cyclopiazonic acid (α-cyclopiazonic acid; CPA, Figure 1) is an indole-tetramic acid mycotoxin produced by the ubiquitous genera of molds *Aspergillus* and *Penicillium*. Beside colonizing various grains and seeds [1,2], these molds can grow on many food substrates, such as cheese and meat products [3,4,5]. Therefore, CPA can contaminate a number of agricultural commodities, animal feeds, and food sources. This toxin has been found in edible tissue in poultry, milk, and eggs [6,7,8] presumptively due to animals’ consumption of contaminated feeds. Despite the wide presence of CPA, few incidents of animal mycotoxicosis and no confirmed incident of human poisoning have been attributed to CPA. Despite its early discovery, the benign nature of CPA has rendered it to receive much less attention of the mycotoxin research community than its counterparts such as aflatoxins, trichothecenes, fumonisins and ochratoxins in the past two decades.

The chemistry and biochemistry of the synthesis of CPA, which was carried out primarily in a *Penicillium* strain, received considerable attention in the 1970s. Some intermediates and enzymes involved in their formation and/or conversions were purified (for a review, see [9] and references therein). The chemical characterization of CPA also has elicited a tremendous interest in studies of biologically active natural products containing the tetramic acid structural motif, an important class of nitrogen-containing heterocycles [10,11,12]. The detailed revelation of CPA formation, especially the aspects of molecular biology and enzymatic mechanisms, however, had been lacking until the recent identification of three clustered biosynthetic genes in *Aspergillus flavus* and closely related *Aspergillus oryzae* [13,14,15]. This review aims to provide an historic overview of CPA studies and the most recent progress made in the elucidation of CPA biosynthesis.

## 2. CPA-Producing Fungi

Cyclopiazonic acid is named after the strain, *Penicillium cyclopium* Westling [16], from which it was originally isolated. However, *P. cyclopium* or its synonym *P. aurantiogriseum* [17] have not been found to make CPA, and the CPA-producing strain originally isolated (CSIR 1082) was later identified as *P. griseofulvum* Dierckx [18]. Other species of *Penicillium* including *P. griseofulvum*, *P. camemberti*, *P. urticae* and *P. commune* have been reported to consistently produce CPA [19]. Other species of *P. chrysogenum*, *P. nalgiovense,P. crustosum*, *P. hirsutum* and *P. viridicatum* also have been reported to produce CPA [20] but CPA production by these taxa has not been confirmed. Certain *Aspergillus* species such as *A. flavus*, *A. oryzae*, *A. fumigatus, A. versicolor,* and *A. tamarii* also produce CPA [21,22,23]. In a recent survey, Vinokurova *et al.* [24] found that 30% of the *A. fumigatus* and *A. phoenicis* strains but only one of 21 *A. versicolor* strains were able to produce CPA. The low incidence of CPA production by *A. versicolor* warrants a further examination since *A. versicolor*  Tiraboschi originally reported to produce CPA [25] was later identified as *A. oryzae* [26].

**Table 1:** Percentage of CPA-producing *A. flavus* isolates from various regions of the world.

Sources	No.^a^	BG^b^	AF/CPA	AF/−	−/CPA	−/−	References
Peanuts, soybean, wheat, Argentina	87	5	27	2	57	14	[27]
Peanuts, Argentina	38	2	79	0	21	0	[28]
Peanuts, Argentina	29	3	49	3	24	24	[29]
Soils, Argentina	218L	8	77	11	11	1	[30]
	73S		88	4	8	0	
	70N		78	9	10	3	
Dried vine berries, Argentina	5		0	0	100	0	[31]
Grain, smoked dried meat products, Croatia	96		0	10	5	85	[32]
Corn, wheat, feeds, Hungary	32		0	0	59	41	[33]
Sour lime, India	25		20	40	?	?	[34]
Soils, Iran	58		21	7	22	50	[35]
Peanuts, Israel	200		19	73	4	4	[36]
Maize, Italy	62	8	45	21	13	21	[37]
Almonds, Portugal	15	1	20	0	0	80	[38]
Feeds, Queensland	31	7	65	3	22	10	[39]
Cocoa beans, Spain	100	20	15	32	17	36	[40]
Maize, US	19		58	5	16	21	[41]
Soils, US	774L		71	<1	12	16	[42]
	309S		99	<1	<1	0	
Corn, nuts, animals and humans, Brazil, Uganda, US	54		26	7	26	41	[43]

a: Number of total strains used in the calculation. L indicates strains that produce large sclerotia; S, small sclerotia and N, no sclerotia.

b: Number of strains that produce B- and G-type aflatoxins not included in the calculation.

CPA and aflatoxins often co-contaminate crops [41,42,44,45,46,47,48]. The source of these mycotoxins is complicated by the fact that both *aspergilli* and *penicillia* are often found in the same crop and as well as after grain storage [49,50,51,52,53]. The most important aflatoxin producers are members in the *Aspergillus* section *Flavi*, particularly *A. flavus*, *A. parasiticus*, and *A. nomius*. Some strains of other members such as *A. pseudotamarii* [54], *A. tamarii*, *A. toxicarius*, and *A. bombycis* [55] also have been reported to produce aflatoxins. *A. flavus* has been divided into two subtypes based on sclerotial size [56,57,58]. The type with small sclerotia (S_B_ or A. *flavus* Group IB) is found together with the L-morphotype (large sclerotia > 400 um) as crop and soil contaminants on all continents except Antarctica. Small sclerotia-producing isolates from crops in Thailand that resemble *A. flavus* S_B_ strains but produce both B- and G- aflatoxins have been called *A. flavus var. parvisclerotigenus* [59]. This taxonomic identification has never been recognized as authoritative, and in an independent investigation of *Aspergillus* section *Flavi* isolates from Thailand certain isolates with small sclerotia and the ability to produce both B- and G- aflatoxins have been identified as a variant clade of *A. nomius* rather than *A. flavus* [60]. Atypical *A. flavus* isolates (also called strain S_BG_ or *A. flavus* Group II), which produce small sclerotia and are intermediate between *A. flavus* and *A. parasiticus* have been identified in soil and agricultural samples from West Africa, Argentina and Australia [28,58,61]. These S_BG_ isolates have recently been classified as *A. minisclerotigenes* and *A. arachidicola* [62]. Only *A. minisclerotigenes* produces CPA. Isolates of these new taxa are also possibly found in the United States (Texas) [58,61] and Europe (Italy, Portugal, and Spain) [37,38,40]. Since the original finding of CPA production by *A. flavus* [63], the relationship between CPA and aflatoxin production by *A. flavus* and the closely related *A*. *parasiticus* has been investigated. Typical *A. flavus* isolates can produce only B-type aflatoxins and CPA, and *A. parasiticus* always produces B- and G-type aflatoxins but never CPA [23]. Table 1 summarizes the ability of *A. flavus* isolates to produce CPA and aflatoxins from various regions of the world. The atypical B- and G-type producers are excluded in Table 1. As can be seen, the ability of *A. flavus* isolates to produce CPA varies greatly. The incidence of CPA production by *A. flavus* is high (>70%) in Argentina and in the United States (Table 1). 

## 3. Toxicological and Pathological Effects of CPA

The toxicity of CPA is attributed to its ability to alter normal intracellular calcium flux through the specific inhibition of sarcoplasmic or endoplasmic reticulum calcium-dependent ATPase (SERCA) essential for calcium uptake as in the muscle contraction-relaxation cycle, which results in increased muscle contraction [64,65]. SERCA also plays a crucial role in other body cells. It serves a housekeeping function, maintaining high calcium in the endoplasmic reticulum and low cytosolic calcium. This calcium gradient is vital for the cell and controls proliferation, differentiation and cell death. Most recent evidence shows that CPA inhibits the calcium pump by blocking the calcium access channel and immobilizing a subset of four transmembrane helices of the ATPase [66]. Besides the specific inhibition of ATPase activity, CPA induced various pathological lesions in test animals [19]. 

In rats, intraperitoneal injection of CPA has been reported to cause lesions of the liver, kidney, pancreas, spleen, and heart [67]. The oral LD_50_ value for CPA in nonpregnant mice was 64 mg/kg body weight, and the toxicity syndromes included ptosis, hypokinesia, hypothermia, tremor, cessation of food and water intake and resulting cachexia. The intensity of these toxic syndromes was dose-dependent. In pregnant mice, a single oral pulse dose of CPA (15 to 50 mg/kg) decreased body weight gain and the pregnancy rates significantly [68].In feeding studies broiler chickens fed 100 ppm of CPA had a high mortality rate and decreased weight gain. Postmortem examination showed that the chickens had proventricular lesions characterized by mucosal erosion and hyperemia, yellow foci in livers and spleens. Microscopic examination of tissues showed ulcerative proventriculitis, mucosal necrosis in the gizzard, and hepatic and splenic necrosis and inflammation. Chickens fed 50 ppm CPA had thick mucosa and dilated proventricular lumens as well as hyperplasia of the proventricular mucosal epithelium. CPA at 10 ppm, however, did not cause significant lesions [22]. Dogs administered CPA at 0.5 mg/kg or 1.0 mg/kg died before the end of 90-day study period, which suggests a much lower no-observable-effect level (NOEL) than other small animals. Gross lesions of diffuse hyperemia with focal areas of hemorrhage and ulceration and microscopic lesions of necrosis, vasculitis, lymphoid necrosis, and karyomegaly in organs were apparent [69]. Surprisingly, among the large mammals, pigs appear to be quite sensitive to CPA with a NOEL of approximately 1.0 mg/kg [70].

The effects of CPA (50 mg/kg) and aflatoxin (3.5 mg/kg) on broiler chickens, in most cases, were additive [71]. Chickens treated with CPA and aflatoxin had increased weights of the liver, kidney, and pancreas, showed signs of proventriculitis, had decreased concentrations of serum albumin and phosphorus, and increased concentrations of blood urea nitrogen and serum glutamic oxalacetic transaminase. Postmortem examination showed that the chickens fed CPA and aflatoxin exhibited similar pathological effects as those fed CPA alone, which included thickened mucosa and dilated proventricular lumens, hard fibrotic spleen, and atrophy of the gizzard.

The IC_50_ of CPA on cell proliferation was 40-fold lower than ochratoxin (OTA) in an *in vitro* test using purified lymphocytes from piglets [72]. The combined effect of CPA and OTA based on IC_20_ on the proliferation of porcine lymphocytes was additive [73]. The individual and combined effect of CPA (34 mg/kg) and OTA (2.5 mg/kg) to broiler chickens were examined [74]. CPA reduced body weight to about 80% of the control, but it did not affect the relative weight of liver, kidney, pancreas except for proventriculus. The interaction of CPA and OTA was additive. The pathological lesions were similar to those caused by CPA and aflatoxin. Animals had increased weights of liver, kidney, pancreas, and proventriculus, decreased serum total protein and albumin, and increased triglycerides, uric acid and creatine kinase activity; creatine is important in maintaining the ATP level for skeletal muscle contraction. Postmortem examination revealed similar pathological lesions as those caused by CPA and aflatoxin.

The feeding of CPA (34 mg/kg) and T-2 toxin (6 mg/kg) also caused reduced performance and adversely affected the health of broiler chickens [75]. A synergistic interaction between CPA and T-2 for relative liver and kidney weights, serum cholesterol and triglyceride concentrations was observed. This combination also affected the 3-week body weight significantly. CPA and T-2 can induce cell death in lymphoid organs [76]. The immunopathological effect of 10 ppm CPA and 1 ppm T-2 in combination included decreases in both thymic CD_8_^+^ lymphocyte and splenic CD_4_^+^ and CD_8_^+^ lymphocytes [77].

CPA is not a potent acute toxin because its oral LD_50_ in rats is in the range of 30 to 70 mg/kg [68,78]. Few incidents of animal mycotoxicosis have been reported because of the benign nature of CPA, the small amounts usually present, or the fact its effects may be masked by other concurrent mycotoxins. Both CPA and aflatoxins were isolated from peanut meal related to the turkey ‘X’ disease that caused the death of 100,000 turkeys in England in the early 1960s [79,80]. Although aflatoxins were regarded as the main culprit, CPA likely contributed to some of the observed pathological clinical signs, such as catarrhal or haemorrhagic enteritis and opisthotonus that are not characteristic of aflatoxicosis [79,81].

Despite the CPA-induced pathological effects in test animals, field incidents of CPA mycotoxicoses in humans have not been reported. Nonetheless, it has been speculated that CPA may be associated with ‘kodo poisoning,’ a toxic reaction characterized by nausea, vomiting, depression, intoxication and unconsciousness after consumption of CPA-contaminated Kodo millet in some parts of North India [78,82]. Based on a NOEL of 1 mg/kg/day and taking into consideration species variability, for humans approximately 10 μg/kg/day or 700 μg/day was suggested as the maximum acceptable daily intake (ADI) dose [19].

## 4. Biosynthesis of CPA

Chemically, CPA belongs to the family of indole-tetramic acid secondary metabolites. *P. griseofulvum* Dierckx (originally called *P. cyclopium*) showed the maximal rate of CPA production after cessation of growth in Czapek’s medium. Research to elucidate the mechanism of CPA biosynthesis was carried out primarily in this strain in the 1970s. The origin of the carbon skeleton of CPA was established by feeding studies using radiolabeled substrates [83]. Chemical degradation analysis indicated that CPA is derived from tryptophan, a C5-unit formed from mevalonic acid and two molecules of acetic acid. The incorporation efficiencies of sodium [1-^14^C]acetate, [2-^14^C] mevalonic acid, and indole-labeled tryptophan were 3.5%, 7.0%, and 24.7%, respectively. The efficient incorporation of labeled tryptophan strongly suggested that it is a direct precursor of CPA. Further studies showed that all carbon atoms of the tryptophan were incorporated into CPA [84]. The presence of both β-CPA (bis-secodehydrocyclopiazonic acid) and CPA in mycelia suggested that β-CPA may be a precursor [85]. As further evidence for β-CPA being the precursor, the time-course of metabolite production showed that, when only trace amounts of CPA were produced, β-CPA predominated, while as CPA formation increased, the amount of β-CPA declined. After feeding [1-^14^C]acetate-labeled β-CPA 67% was converted to CPA, while 18% remained as β-CPA [83]. The compound *γ,γ*-dimethylallyltryptophan was known to be a precursor of ergot alkaloids [86,87,88]. Incubation of a cell-free-extract (CFE) with a mixture of radiolabeled [^3^H]tryptophan and [1-^14^C] dimethylallyl diphosphate (DMAPP) did not yield CPA labeled with either ^3^H or ^14^C although the resulting β-CPA contained 19% of the added ^14^C, but no ^3^H [89]. This result showed that the labeled DMAPP, but not tryptophan could be incorporated into β-CPA by the CFE. This study showed that CPA formation was different from ergot alkaloid biosynthesis, in the latter biosynthesis involves direct prenylation of tryptophan by DMAPP [86,90]. Subsequent studies found that the substrate for DMAPP in the CFE was α-acetyl-γ-(β-indolyl)methyltetramic acid (cycloacetoacetyl-L-tryptophan, cAATrp) [84]. The CFE converted exogenously supplied cAATrp and DMAPP into β-CPA in 1:1 stoichiometry. The responsible enzyme in the CFE was designated as cycloacetoacetyltyptophanyl dimethylallyl transferase. This enzyme, also called β-CPA synthase or dimethylallyl cycloacetoacetyl typtophan synthase (DCAT-S) was purified by McGrath *et al.* (1977). It has a molecular weight of 95 kDa with an isoelectric point of 5.3. Its concentration reached a maximum at 60 hr during fermentation. This synthase is very specific for DMAPP and cAATrp and could not use isopentenyl diphosphate (IPP, an isomer of DMAPP), tryptophan or *N*-acetyltryptophan as substrates [91]. The dimethylallyl tryptophan synthases of *Clavices purpurea*, *Aspergillus fumigatus, Aspergillus nidulans* and *Malbranchea aurantiaca* for alkaloid biosynthesis are α_2_ dimers [86,92] suggesting that the β-CPA synthase is also likely to be a dimer. The enzyme(s) for the formation of cAATrp was never isolated from *P. griseofulvum* Dierckx. Schabort *et al.* [93] isolated five isozymes, designated as β-cyclopiazonate oxidocyclase that catalyzed oxidation and cyclization of β-CPA to CPA; each of the β-cyclopiazonate oxidocylases has a covalently linked flavin. Spectrophotometric titration studies of oxidized β-cyclopiazonate oxidocyclase with substrate analogs showed that the NH group in the tetramic acid residue is involved in binding to the active site [94,95].

### 4.1. The mevalonate pathway key to CPA formation in A. flavus

**Scheme 1:**
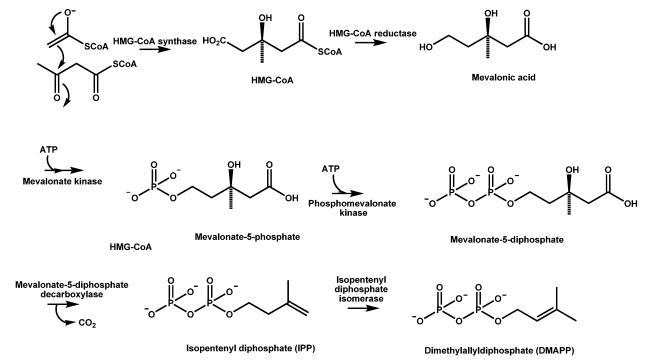
Biosynthesis of dimethylallyldiphosphate (DMAPP).

Mevalonate is the primary precursor of DMAPP, which is also a major component in the production of terpenoids. The biosynthetic steps leading to the formation of mevalonic acid have been well characterized [96] and are shown in Scheme 1. They include condensation of acetyl-CoA and acetoacetyl-CoA by 3-hydroxy-3-methylglutaryl (HMG)-CoA synthase and a subsequent reduction by HMG-CoA reductase.In eukaryotes, conversion of mevalonic acid to DMAPP involves two consecutive phosphorylations at position C5 by mevalonate kinase and phosphomevalonate kinase, respectively [97]. The subsequent decarboxylation carried out by diphosphomevalonate decarboxylase (or mevalonate-5-diphosphate decarboxylase), followed by dehydration produces isopentenyl diphosphate (IPP). IPP is a common intermediate for a number of pathways including those for the synthesis of terpenoids, steroids and carotenoids, and prenyl moieties of sirodesmin and ergot alkaloids. IPP is then isomerized by IPP isomerase to DMAPP, a potent electrophile [98]. DMAPP can alkylate other molecules, including IPP, to generate a variety of isoprenoid compounds found in nature such as plant hormones, mycotoxins, antimicrobial agents, and pharmaceutically important antibiotics. 

The availability of full genome sequences for many organisms makes it possible to locate physically linked genes in a genome and to quickly obtain gene copy number. The genome sequences of *A. oryzae* RIB40 (ATCC 42149) and *A. flavus* NRRL3357 are available for such analysis at http://www.bio.nite.go.jp/dogan/Top and http://www.aspergillusflavus.org/genomics/, respectively. Both genomes are about 37 Mb and organized in eight chromosomes [99,100]. These genome databases provide a remarkable wealth of information about identity, function, and physical location of many genes. Candidate genes involved in the anabolism and catabolism of mevalonic acid thus can be readily identified from respective genome databases (Table 2). The genes encoding key enzymes in DMAPP biosynthesis are not clustered and appear as multiple copies. There are two copies of genes encoding 3-hydroxy-3-methylglutaryl (HMG)-CoA synthase and HMG-CoA reductase and one copy each of genes encoding mevalonate kinase, phosphomevalonate kinase, and diphosphomevalonate decarboxylase. The predicted enzymes contain a conserved Gly/Ser-rich region probably involved in the binding of ATP known as a GHMP (abbreviations from their substrates, galacto-, homoserine, mevalonate and phosphomevalonate) kinase N terminal domain as well as a GHMP kinase C terminal domain. The mevalonate kinase gene and the diphosphomevalonate decarboxylase gene are about 190 kb apart on chromosome 3, and the phosphomevalonate kinase gene is 50 kb from one of the two HMG-CoA synthase genes on chromosome 8. The two IPP isomerase genes are located on chromosome 3 and about 300 kb apart. 

**Table 2:** Predicted loci associated with the metabolism of mevalonic acid.

Enzyme	Gene Locus^a^	Location
`3-Hydroxy-3-methylglutaryl-CoA synthase	AFL2G_02388.2	Contig 2, 2508858~2510466 −
	AFL2G_11649.2	Contig 14, 1056212~1057748 −
	AO090003000611	Chromosome 2, SC003,1640889~1642497 +
	AO090010000487	Chromosome 8, SC010, 1307684~1309220 −
HMG-CoA reductase	AFL2G_08234.2	Contig 9, 779011~783044 -
	AFL2G_12082.2	Contig 15, 462348~465804 -
	AO090120000217	Chromosome 5, SC113, 541346~544055 -
	AO090103000311	Chromosome 8, SC103, 805908~807609 +
Mevalonate kinase	AFL2G_04604.2	Contig 4, 2076279~2077883 −
	AO090023000793	Chromosome 3, SC023, 2058446~2060050 -
Phosphomevalonate kinase	AFL2G_11633.2	Contig 14, 1009373~1011005 -
	AO090010000471	Chromosome 8, SC010, 1260354~1261854 -
Pyrophosphomevalonate decarboxylase	AFL2G_04673.2	Contig 4: 2266676~2268145 -
	AO090023000862	Chromosome 3, SC023, 2250974~2252258 −
Isopentenyl pyrophosphate isomerase	AFL2G_04341.2	Contig 4, 1322573~1323720 +
	AFL2G_04245.2	Contig 4: 1026352~1027394 +
	AO090023000500	Chromosome 3, SC023, 1306204~1307211 +
	AO090023000391	Chromosome 3, SC023, 1004129~1005046 +

a: AFL2G indicates *A. flavus* genes and AO indicates *A. oryzae* genes.

### 4.2. The gene cluster involved in CPA biosynthesis is adjacent to the aflatoxin gene cluster in A. flavus/oryzae

The aflatoxin gene cluster of *A. flavus* NRRL3357resides in a subtelomeric region on chromosome 3. Even before the genome sequences of *A. oryzae* and *A. flavus* became available it was suspected that the genes involved in CPA production are located close to the aflatoxin gene cluster because many *A. flavus* strains unable to produce aflatoxins also did not produce CPA. Further analysis found that such isolates lost large portions of or the entire aflatoxin gene cluster up to the telomere [101]. *A. oryzae* is closely related to *A. flavus,* and some strains also produce CPA [15]. In the effort to generate *A. oryzae* mutants that do not produce aflatoxins, CPA, kojic acid, and 3-nitropropionic acid for producing polypeptides, Christensen *et al.* [102] gamma-irradiated *A. oryzae* BZ14, an isolate derived from *A. oryzae* IFO 04177, which contains the aflatoxin gene cluster. They obtained two CPA-negative mutants, BECh1 and BECh 2, which lost the aflatoxin pathway *aflR* and *omtA* genes. Because *aflR* and *omtA* are 22 kb apart in the genomes of *A. flavus* and *A. parasiticus*, gamma radiation treatment must have caused deletion of a large portion of DNA containing genes encoding proteins involved in aflatoxin biosynthesis and CPA biosynthesis. From this study it was suspected that part of the deleted region is associated with CPA production. Christensen *et al.* [103] later isolated the dimethylallyl-cycloacetoacetyl-L-tryptophan synthase (DCAT-S) gene from an *A. oryzae* A1560 (=IFO 04177) isolate. Disruption of this gene resulted in the loss of CPA production.

**Figure 2:**
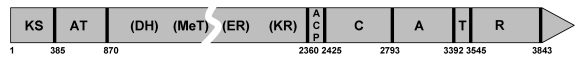
Schematic representation of the PKS-NRPS involved in synthesis of cAATrp. The different domains are shown in the arrow (KS, ketosynthase; AT, acyl transferase; DH, dehydratase; MeT, methyltransferase; ER, enoyl reductase; KR, ketoreductase; ACP, acyl carrier protein attachment site; C, condensation domain; A, adenylation domain; T, thiolation domain; R, releasing domain). Brackets indicate the domains are defective. The numbers below the arrow indicate approximation amino acid distance from the *N*-terminus for the domains.

**Figure 3:**
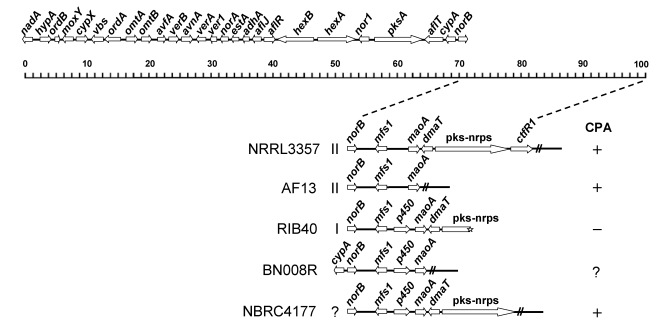
Partial and complete CPA clusters in different strains of *A. flavus* and *A. oryzae*. The AF biosynthesis cluster is shown above the scale (kb).

In *A. oryzae* RIB40, which is unable to produce CPA, the aflatoxin gene cluster boundary gene *norB* is about 19 kb from the telomere at one end on chromosome 3 (Figure 3) suggesting that the DCAT-S gene is located between the aflatoxin gene cluster and the telomere. In the genome of *A. oryzae* NBRC 4177 (=IFO 04177), a CPA-producing strain, an additional region is present extending beyond the telomere in the RIB40 strain (Figure 3). Tokuoka *et al.* [15], using PCR profiling with oligoprimers derived from the same region in the *A. flavus* NRRL3357 genome sequence combined with an inverse PCR technique, identified the telomeric repeat and determined that the size of the region in *A. oryzae* NBRC 4177 extending beyond the telomere of *A. oryzae* RIB40 is about 17 kb. Sequencing of the region around the RIB40 telomere in *A. oryzae* NBRC 4177 revealed a gene predicted to encode a hybrid polyketide synthase (PKS)-nonribosomal peptide synthase (NRPS). This *pks-nrps* gene is 11.7 kb and lacks introns. In addition, other genes in this region include: *cpaB* (encoding the DCAT-S), *cpaC,* predicted to encode a cytochrome P450 monooxygenase, a gene possibly encoding a methyltransferase, and *cpaT,* predicted to encode a transporter. As with other secondary metabolite gene clusters, clustering of genes may allow efficient coordinated regulation of gene expression when CPA production is necessary. 

Extensive PCR profiling of the 100-kb subtelomeric region of *A. flavus* strains of various chemotypes and closely related *A. parasiticus* strains, which produce aflatoxins, but not CPA, suggested that a CPA biosynthesis cluster is next to the aflatoxin gene cluster [13]. Four genes were characterized in this putative CPA gene cluster by gene disruption: *maoA*, predicted to encode a monoamine synthase, *dmaT*,the gene encoding the dimethylallyltryptophan synthase,  *pks-nrps*, and a Zn_2_Cys_6_ transcription factor-encoding gene, *ctfR1* (Figure 3). Consistent with studies performed on *A. oryzae*, disruption of *dmaT* (=the DCAT-S gene) and *pks-nrps* abolished CPA production [15,102]. Knockout studies confirmed that the gene *maoA* is also required for CPA biosynthesis. Surprisingly, disruption of *ctfR1*, which was expected to be required for transcriptional regulation of the CPA cluster genes, did not affect CPA production. A 3-kb region between the aflatoxin gene cluster and *maoA* in *A. flavus* did not contain ORFs likely to encode proteins related to CPA biosynthesis (see Figure 3). Therefore, *maoA*, *dmaT*, *pks-nrps* constitute the CPA gene cluster in *A. flavus*. A search of the putative promoter region for the *pks-nrps* for potential Zn_2_Cys_6_ protein binding sites revealed a sequence, 5’-TCGTGGACGA-3’, which is quite similar to AflR-binding sites [104]. If AflR is acting as the pathway specific regulatory protein for both the aflatoxin and the CPA gene clusters, this would explain their apparent co-regulation under similar media conditions and the reason why proximity to the aflatoxin gene cluster is maintained.

## 5. Characterization of the Enzymes Involved in CPA Biosynthesis

### 5.1. Formation of cAATrp by CpaS, a hybrid PKS-NRPS

**Scheme 2:**
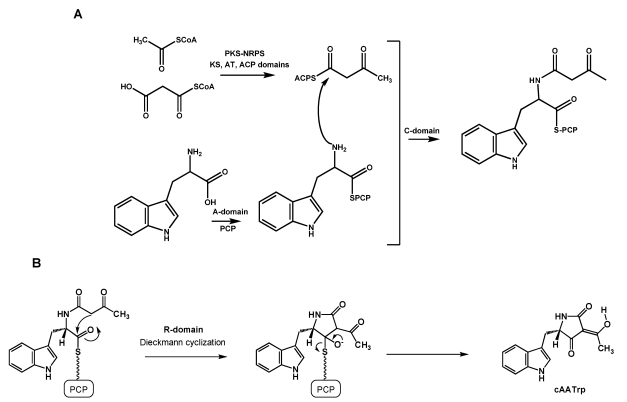
Conversion of acetylCoA to cAATrp by the hybrid PKS-NRPS. A. Formation of the cAATrp precursor catalyzed by the KS, AT, ACP, A, and C domains. B. Dieckmann cyclization to form cAATrp catalyzed by the R domain.

Formation of the first stable intermediate in CPA biosynthesis, *cyclo*-acetoacetyl-L-tryptophan (cAATrp), in *A. flavus/oryzae* requires catalysis by the 431 kDa multifunctional CPA synthase (CpaS or CpaA), a PKS/NRPS polypeptide with seven functional and four non-functional catalytic domains [14,15] (Figure 2) The PKS portion of CpaS contains three functional domains, a ketosynthase (KS), an acyl transferase (AT), and an acyl carrier protein (ACP) domains that catalyze formation of acetoacetyl-CoA by condensation of one molecule of acetyl-CoA with one molecule of malonyl-CoA. Domains of dehydratase (DH), methyltransferase (MT), enoylreductase (ER) and ketoreductase are present in the CpaS but lack critical amino acids necessary for catalytic activity [14]. After condensation of two molecules of acetate by the KS, the acetoacetyl moiety is tethered on ACP. The NRPS portion of CpaS shown in Figure 2 has catalytic domains common to many NRPSs, including a condensation domain (C, amino acids 2501-2793), an adenylation domain (A, amino acids 2980-3392) to activate an amino acid for loading, a thiolation domain (T, amino acids 3481-3546) that is an attachment site of the growing peptide on the phosphopantetheinylated peptidyl carrier protein (PCP), and a releasing domain (R, amino acids 3593-3619) to release the product peptide from the enzyme. The biosynthesis steps leading to cAATrp are shown in Scheme 2.

Amino acid selection by the A domain is dependent on signature amino acids in the domain that have not been well defined [105]. However a tool is available that predicts whether or not large (aromatic amino acids) or small amino acids will be accepted by the A domain [106]. Prediction for CpaS is that large amino acids such as Trp or Phe will be recognized by its A-domain. How Trp rather than Phe is recognized by the CpaS A-domain has not been determined. During synthesis of cAATrp, the adenylation domain activates the substrate Trp which is then transferred to the pantetheine cofactor of the neighboring peptidyl carrier protein (PCP) domain (the T domain in Figure 2) [107]. 

Gulick [105], in reviewing the required conformation dynamics for catalysis by NRPSs, found evidence for a domain alternation strategy between the A and T domains that consists of two catalytic steps: the first involving the initial adenylation of the amino acid carboxylate moiety to form an acyl-AMP intermediate and the second involving formation of the thioester. He suggested that the enzymes use a 140° domain rotation to present opposing faces of the two domains to the active site in order to allow both partial reactions. The crystal structure of a four-domain NRPS SrfA-C protein, which is similar to CpaS, was determined and demonstrated that the A domain is rotated away from the *N*-terminal T domain, thereby allowing the PCP domain to interact with the neighboring C domain. The PCP domain is not positioned so that it could donate the pantetheine arm to either the A domain or the R domain.

The C domains of NRPSs are known to catalyze amide bond formation between two PCP-tethered amino acids [108,109]. For CpaS, condensation occurs between the ACP-tethered acetoacetyl moiety and the PCP-tethered tryptophan and forms an amide bond. Condensation requires the involvement of the highly conserved HHXXXDX_14_Y motif found in all modular C domains [110]. The Asp and second His were found to be essential for catalytic activity. Following condensation the substrate remains tethered to the PCP phosphopantetheinyl arm in the T domain.

Comparisons of both hybrid PKS-NRPS and NRPS enzymes show that the most downstream domain of the NRPS module acts as the chain-terminating module. Therefore, the release of the condensation product acetoacetyl-Trp is likely to be mediated by such an R domain. The role of the R domain in the release of the metabolite from the PKS-NRPS has recently been found to occur without the expected NADH/NADPH reduction even though the R domain contains an NADH/NADPH binding domain (GXXG/SXXG) near its *N*-terminus [111]. However, the domain lacks a functional short chain dehydrogenase/reductase (SDR) catalytic triad (Ser-Tyr-Lys) characteristic of similar NRPSs and other enzymes (aldehyde reductase, epimerases) known to use such domains for thioester and ester hydrolysis. In CpaS, the Tyr is replaced by Leu in this triad which would prevent catalytic function as a reductase. Other amino acids further toward the *C*-terminus of the R-domain were found to be necessary for catalysis, in particular, Asp3803 and His3843. The His residue could be involved in proton abstraction necessary to form the resonance-stabilized acetoacyl carbanion shown in Scheme 2A. It is this carbanion that attacks the thioester carbonyl by Dieckmann condensation to cyclize acetoacetyl-Trp and release it from PCP to form the indole tetramate, cAATrp. 

### 5.2. Conversion of cAATrp to β-CPA

**Scheme 3:**
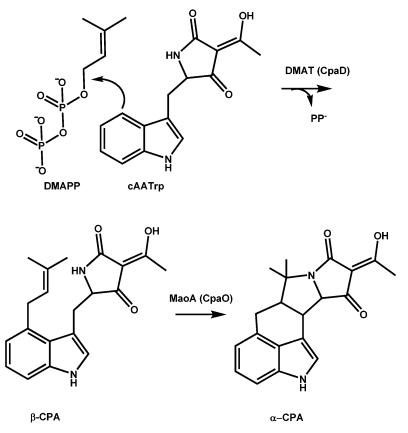
Conversion of cAATrp to α‑CPA.

Conversion of cAATrp to β-CPA (Scheme 3) involves alkylation of the C-4 position of the Trp indole ring by DMAPP, a reaction catalyzed by cycloacetoacetyltyptophanyl dimethylallyl transferase [91] encoded by the CPA-cluster gene, *dmaT*. The transferase also is called dimethylallyl cycloacetoacetyl typtophan synthase (DCAT-S) or designated as CpaD [111]. *A. flavus* and *A. oryzae* DMATs consist of 437 amino acids with a calculated molecular weight of 49 kDa, and the functional form likely is a dimer as reported for many dimethylallyl tryptophan synthases involved in the synthesis of alkaloids. There are at least nine *dmaT* homologs in the *A. flavus* genome, but only the *dmaT* in the CPA gene cluster is associated with a *pks-nrps* gene. 

Involvement of dimethylallyl tryptophan synthases (DMATs) in other biosynthetic processes has been well documented. Some of these DMATs may be used in synthesis of alkaloids related to ergot, where the first committed step in the biosynthesis is tryptophan prenylation. Since disruption of the CPA-cluster *dmaT* prevents CPA biosynthesis, the DMAT in the CPA cluster appears to be the only DMAT able to catalyze prenylation of cAATrp. The presence of *dmaT* in the CPA gene cluster supports the assembly-line nature of β-CPA biosynthesis similar to those characterized for NRPSs in prokaryotes. Catalytic activity has recently been studied by analyzing the crystal structure of a complex of *A. fumigatus* DMAT with L-tryptophan [112]. 

The protein forms a β/α barrel fold called a prenyltransferase barrel. Enzymes with similar structure are found in bacteria but have no sequence similarity to fungal DMATs. The crystal structure revealed that dimethylallyl diphosphate is positioned selectively in the enzyme over position 4 of the Trp indole nucleus (Scheme 3). The reaction catalyzed by DMAT is an electrophilic alkylation, (Friedel-Crafts alkylation), of an aromatic ring. The energy barrier for cation formation that precedes the alkylation is overcome by tight interactions with Arg and Lys residues in the catalytic pocket that offset the diphosphate negative charges. The resulting carbocation is stabilized by five Tyr residues to provide a hydrophobic pocket that prevents reaction of the carbocation with water. Just as in a typical Friedel-Crafts reaction Mg^2+^ facilitates the alkylation.

### 5.3. Conversion of β-CPA to α-CPA is catalyzed by the monoamine oxidase MaoA (CpaO)

The final step in the biosynthesis of α-CPA is the dehydrogenation of β-CPA resulting in intramolecular ring closure (Scheme 3, bottom). The enzyme involved in this conversion (CpaO) is a FAD-dependent oxidoreductase that has some of the properties of typical monoamine oxidases. Early studies suggested that the CPA oxidoreductase first generates an unstable eneimine which then undergoes intramolecular cyclization [94,95,113,114]. The role of the flavin prosthetic group in catalysis by FAD-dependent oxidases has been the subject of much study and although the ring-closure appears surprisingly simple the mechanism for the catalysis is still under scrutiny [115,116,117]. The deprotonated (rather than the protonated) amine moiety on the substrate initially binds to the active site on the monoaminoxidase and is oxidized to the protonated imine with the covalently bound FAD cofactor being reduced to its hydroquinone form. To complete the catalytic cycle, the reduced FAD cofactor reacts with O_2_ to generate oxidized flavin and H_2_O_2_. Characterized monoamine oxidases exist as membrane-bound dimeric proteins. In human monoamine oxidase A (MaoA), substrate binding and oxidation occur in elongated cavities extending from the flavin site at the core of the enzyme to the surface of the protein on the opposite side of the FAD adenosine ring. From crystal structure determinations, the cavity of the enzyme is hydrophobic. C-H bond cleavage is catalyzed by monoamine oxidases to form the protonated imine; the cleavage could involve transfer of a proton, a hydride or a hydrogen atom to the flavin. 

**Scheme 4:**
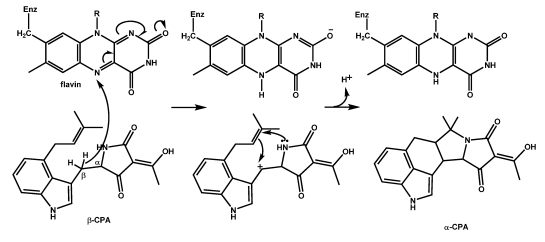
Possible mechanism for oxidative ring-closure of β-CPA to α-CPA by the flavin-dependent monoamine oxidase, MaoA (CpaO).

Unlike the usual oxidations catalyzed by monamine oxidases which involve proton transfer to the flavin [116], the oxidation of β-CPA by CpaO could be initiated by a hydride shift mechanism similar to that postulated for cyclizations catalyzed by the berberine bridging enzyme oxidases [116,118,119,120]. The steps most likely involve hydride transfer to the N4-flavin nitrogen to generate a stabilized cation that undergoes the ring closure as shown in Scheme 4. A radical intermediate could just as easily explain the ring closure steps. However, Edmondson *et al.* [116] suggested that a polar mechanism for monoamine oxidase catalysis fits theoretical calculations best. Formation of an unstable eneimine intermediate as previously suggested [95] is unlikely and ring formation probably occurs immediately upon hydride transfer without accumulation of an intermediate. 

## 6. Genetic Diversity in CPA Biosynthesis of *A. flavus* and *A. oryzae*

The gene preceding *maoA* of the CPA gene cluster in *A. oryzae* RIB40 and NBRC 4177 encodes a P450 monooxygenase while in the same relative location in *A. flavus* NRRL3357 and AF13 no such a gene is present (Figure 3). The unique location of this *p450* gene (=*cpaC*) in *A. oryzae* is reminiscent of the same gene organization in the *Aspergillus* S_BG_ isolate BN008R (ATCC MYA379) collected from Benin, West Africa [61,121]. A comparison of the 12 kb region beyond the *norB* gene in BN008R to the corresponding region in RIB40 shows that they share > 95% nucleotide identity (>98% in the *maoA* and the *p450* coding regions). *A. oryzae* mutants with *p450* deleted failed to produce mono- and dihydroxy CPA when compared to the wild type [122]. Since the *p450* gene is missing in CPA-producing *A. flavus* NRRL3357 and AF13 (Figure 3), it is not directly involved in CPA biosynthesis. It is not known whether *A. oryzae* RIB40 gained the *p450* gene or *A. flavus* NRRL3357 lost the *p450* gene.

RIB40 and NRRL3357 exhibit other unique differences; the former has the type I deletion in its *norB-cypA* region, an S-strain signature, while the latter has the type II deletion that is often associated with aflatoxigenic L-strain *A. flavus* isolates [123]. They also have distinct single nucleotide polymorphisms in the *omtA* and *dmaT* genes involved in aflatoxin and CPA biosynthesis, respectively [58,123] (and unpublished results). *A. flavus* populations are genetically diverse. *A. oryzae* is believed to be selected or to have evolved from certain groups of nonaflatoxigenic *A. flavus*. *A. oryzae* strains are classified into three groups based on deletion patterns in the aflatoxin gene cluster [124,125]. Group 1 has all aflatoxin gene orthologs, group 2 has the region beyond the *ver1* gene deleted but contains a DNA segment of unknown origin, and group 3 has the partial aflatoxin gene cluster up to the *vbs* gene (see Figure 3). Group 3 resembles the E, F, and G deletion patterns in nonaflatoxigenic and CPA-negative *A. flavus* isolates [101], which lack the genome region beyond the respective partial aflatoxin gene cluster (connected to telomeric repeat). Since the CPA gene cluster in *A. oryzae* resides next to the aflatoxin gene cluster as in *A. flavus*, group 3 likely has lost the CPA gene cluster. Group 2 strain RIB62 has been confirmed to be lacking the CPA gene cluster resulting from a large deletion [125]. Some strains of group 1 *A. oryzae* still maintain the ability to produce CPA, such as NBRC 4177 while other strains have lost the ability, such as RIB40 due to a truncation that caused the loss of more than half of the *pks-nrps* gene.

A PCR survey of the strains of *A. oryzae* and *A. flavus* and related *A. parasiticus* and *A. sojae* (Table 3) showed that only isolates of nonaflatoxigenic and CPA-positive L-strain *A. flavus*, with the type I, S-strain genetic signature in a previously characterized clade, for example, MS1-1, NC3-6, SC3-5 and TX21-9 [123] also have the *p450* gene. 

**Table 3:** Sclerotial, genetic, and toxin-producing characteristics of isolates of *Aspergillus* species.

*Species*	*Strain*	*Sclerotial Morphotype^a^*	*AF/CPA Production^b^*	*norB-cypA Pattern^c^*	*p450^d^*
A. flavus	CA28	S	+/+	I	N
	CA42	S	+/+	I	N
	CA43	S	+/+	I	N
	CA44	S	+/+	I	N
	AF12	S	+/+	I	N
	AF70	S	+/+	I	N
	GA10-18	S	+/+	I	N
	VA4-36	S	+/+	I	N
	AF13	L	+/+	II	N
	CA14	L	+/+	II	N
	CA19	L	+/+	II	N
	GA9-9	L	+/+	II	N
	GA13-9	L	+/+	II	N
	NRRL3357	L	+/+	II	N
	VA2-9	L	+/+	II	N
	LA4-5	L	−/+	I	N
	SC6-9	L	−/+	I	N
	TX9-8	L	−/+	I	N
	GA4-4	L	−/+	II	N
	LA10-4	L	−/+	II	N
	MS1-1	L	−/+	I	Y
	NC3-6	L	−/+	I	Y
	SC3-5	L	−/+	I	Y
	TX21-9	L	−/+	I	Y
A. oryzae	NBRC 4177	?	−/+	?	Y
	RIB40	N	−/−	I	Y
	SRRC304	N	−/?	I	Y
	SRRC493	N	−/?	I	Y
	SRRC2044	N	−/?	I	Y
A. parasiticus	BN009	L	+/−	intact	N
	SRRC2043	L	+*/−	intact	N
	SRRC2999	L	+/−	intact	N
A. sojae	SRRC299	N	−/?	intact	N
	SRRC1123	N	−/?	intact	N
	SRRC1126	N	−/?	intact	N

a: S and L indicate S-strain and L-strain isolates based on the size of sclerotia produced.

b: I and II indicate type I and type II deletions in the *norB-cypA* region.

c: The *p450*-specific oligonucleotides used in PCR, tgtgacggtggatggcgagc and tcaatggctttgtactccag, were derived from identical regions of *A. oryzae* RIB40 and *Aspergillus* S_BG_ strain, BN008R.

As mentioned earlier, atypical *A. flavus* S_BG_ isolates have been classified into the new taxa of *A. minisclerotigenes* and *A. arachidicola* [62]. *A. minisclerotigenes* differs from *A. arachidicola*. in that it produces CPA like *A. parvisclerotigenus*, previously named *A. flavus var. parvisclerotigenus* by Saito and Tsuruta [59], which included largely S_B_ strains and only a small number of S_BG_ strains [56,59]. Genetic evidence shows that the *β*-tubulin gene sequence of the *A. parvisclerotigenus* strain, CBS 121.62^T^, isolated from Nigeria by Hesseltine *et al.* [56], is different from those of the *A. minisclerotigenes* strains and this strain produced other metabolites, such as A30461 and speradine A not produced by *A. minisclerotigenes* [62]. 

Although Saito and Tsuruta [59], in their naming of *A. flavus var. parvisclerotigenus*, included morphologically similar S_BG_ isolates obtained by Hesseltine *et al.* [56], Ehrlich *et al.* [60] considered the S_BG_ isolates collected from different regions of Thailand by Saito and Tsuruta [59] a variant clade of *A. nomius* rather than *A. flavus*. The S_BG_*Aspergillus* BN008R isolated from Benin, West Africa [61] likely is *A. minisclerotigenes*. We hypothesize that the common ancestor of the aforementioned nonaflatoxigenic and CPA-positive L-strain *A. flavus* isolates and the group 1 *A. oryzae* that contain the *p450* gene may be *A. minisclerotigenes* or *A. flavus var. parvisclerotigenus.* The loss of both B- and G-type aflatoxins renders them to be regarded as *A. flavus* due to the morphological and cultural similarities to other clades of *A. flavus*. Probably, two genetic variants are responsible for CPA production in *A. flavus*. One is the S_B_*A. flavus* strains that always have the type I *norB-cypA* deletion, and another is L-strain *A. flavus* strains with the type II deletion, which are either aflatoxigenic or nonaflatoxigenic.

## 7. Possible Advantage of CPA to Fungi

Like aflatoxins, the benefit of CPA to the producing fungi is not clear. CPA is an excellent chelator of iron. CPA production is greatest during the period preceding dormancy when growth has practically stopped. Because concentrations of free iron in soil are usually low, it would be beneficial to fungi to have a readily available supply of iron for subsequent growth. Riley and Goeger [126] speculated that stockpiles of CPA-chelated iron would be conducive to rapid fungal growth for occupying a niche in the saprophytic environments. Similar to a siderophore, which is an iron-chelating compound secreted by bacteria, fungi, and plants, CPA probably fulfilled partly this ion-chelating function long ago before a true siderophore took its place. Many siderophores are nonribosomal peptides and dissolve ions by chelation as soluble Fe^3+^ complexes that are taken up by active transport mechanisms. In the *A. flavus* genome, in the same subtelomeric region where the CPA gene cluster resides, *sidC* and *sidT* (*msf2*) genes encoding a siderophore and a siderochrome-iron transporter, respectively, have been identified [13]. Their location, which is at a terminus of chromosome 3, suggests that they were acquired later than the CPA gene cluster to carry out the iron-chelating function.

## 8. Conclusions

The CPA and aflatoxin gene clusters are contiguous on the subtelomeric region of chromosome 3 in the *A. flavus* and *A. oryzae* genomes. The enzyme functions of the three CPA genes correlate well with current as well as previous studies of the biosynthesis of this indole-tetramic acid product. Although CPA is not a potent acute toxin and few incidents of mycotoxicoses have been reported, aflatoxin B_1_ is a potent carcinogen. Little is known about the synergistic effects of the two toxins and more research is needed especially because plans are being considered to apply nonaflatoxigenic *A. flavus* strains in large amounts to control aflatoxin contamination in corn, cottonseed, and peanuts and some *A. oryzae* strains used in food fermentation are capable of CPA production. With knowledge of the co-localization of the two gene clusters it is now easy to explain why strains of *A. flavus* and *A. oryzae* have different abilities to produce aflatoxin and CPA. This understanding has significant health implications. The genetic diversity of *A. flavus* and *A. oryzae* in the region adjoining the CPA gene cluster suggests a divergence of *A. flavus* from *A. oryzae*. We suggest that *A. oryzae* most likely descended from an ancestor that was the precursor of the *Aspergillus* S_BG_ variant, while *A.flavus* descended from a precursor of *A. parasiticus*.

## References

[1] Abbas H.K., Accinelli C., Zablotowicz R.M., Abel C.A., Bruns H.A., Dong Y., Shier W.T. (2008). Dynamics of mycotoxin and *Aspergillus flavus* levels in aging Bt and non-Bt corn residues under Mississippi no-till conditions. J. Agric. Food Chem..

[2] Njobeh P.B., Dutton M.F., Koch S.H., Chuturgoon A., Stoev S., Seifert K. (2009). Contamination with storage fungi of human food from Cameroon. Int. J. Food Microbiol..

[3] Finoli C., Vecchio A., Galli A., Franzetti L. (1999). Production of cyclopiazonic acid by molds isolated from Taleggio cheese. J. Food Prot..

[4] Sosa M.J., Cordoba J.J., Diaz C., Rodriguez M., Bermudez E., Asensio M.A., Nunez F. (2002). Production of cyclopiazonic acid by *Penicillium commune* isolated from dry-cured ham on a meat extract-based substrate. J. Food. Prot..

[5] Lopez-Diaz T.M., Santos J.A., Garcia-Lopez M.L., Otero A. (2001). Surface mycoflora of a Spanish fermented meat sausage and toxigenicity of *Penicillium* isolates. Int. J. Food Microbiol..

[6] Dorner J.W., Cole R.J., Erlington D.J., Suksupath S., McDowell G.H., Bryden W.L. (1994). Cyclopiazonic acid residues in milk and eggs. J. Agric. Food Chem..

[7] Norred W.P., Porter J.K., Dorner J.W., Cole R.J. (1988). Occurrence of the mycotoxin cyclopiazonic acid in meat after oral administration to chickens. J. Agri. Food Chem..

[8] Oliveira C.A.F., Sebastio L.S., Fagundes H., Rosim R.E., Fernandes A.M. (2008). Aflatoxins and cyclopiazonic acid in feed and milk from dairy farms in Sao Paulo, Brazil. Food Add. Contam..

[9] Holzapfel C.W., Steyn P.S. (1980). The biosynthesis of cyclopizonic acid and related tetramic acids. The Biosynthesis of Mycotoxins.

[10] Spatz J.H., Welsch S.J., Duhaut D., Jäger N., Boursier T., Fredrich M., Allmendinger L., Ross G., Kolb J., Burdack C., Umkehrer M. (2009). Tetramic acid derivatives via Ugi-Dieckmann-reaction. Tetrahedron Lett..

[11] Yang Y.L., Lu C.P., Chen M.Y., Chen K.Y., Wu Y.C., Wu S.H. (2007). Cytotoxic polyketides containing tetramic acid moieties isolated from the fungus *Myceliophthora thermophila*: Elucidation of the relationship between cytotoxicity and stereoconfiguration. Chemistry.

[12] Iwata Y., Maekawara N., Tanino K., Miyashita M. (2005). Tetramic acid antibiotics: Stereoselective synthesis of streptolic acid and tirandalydigin. Angew. Chem. Int. Ed..

[13] Chang P.-K., Horn B.W., Dorner J.W. (2009). Clustered genes involved in cyclopiazonic acid production are next to the aflatoxin biosynthesis gene cluster in *Aspergillus flavus*. Fungal Genet. Biol..

[14] Seshime Y., Juvvadi P.R., Tokuoka M., Koyama Y., Kitamoto K., Ebizuka Y., Fujii I. (2009). Functional expression of the *Aspergillus flavus* PKS-NRPS hybrid CpaA involved in the biosynthesis of cyclopiazonic acid. Bioorg. Med. Chem. Lett..

[15] Tokuoka M., Seshime Y., Fujii I., Kitamoto K., Takahashi T., Koyama Y. (2008). Identification of a novel polyketide synthase-nonribosomal peptide synthetase (PKS-NRPS) gene required for the biosynthesis of cyclopiazonic acid in *Aspergillus oryzae*. Fungal Genet. Biol..

[16] Holzapfel C.W. (1968). The isolation and structure of cyclopiazonic acid, a toxic metabolite of *Penicillium cyclopium* Westling. Tetrahedron.

[17] Hermansen K., Frisvad J.C., Emborg C., Hansen J. (1984). Cyclopiazonic acid production by submerged cultures of *Penicillium* and *Aspergillus* strains. FEMS Microbiol. Lett..

[18] Frisvad J.C. (1989). The connection between the *Penicillia* and *Aspergilli* and mycotoxins with special emphasis on misidentified isolates. Arch. Environ. Contam. Toxicol..

[19] Burdock G.A., Flamm W.G. (2000). Review Article: Safety assessment of the mycotoxin cyclopiazonic acid. Int. J. Toxicol..

[20] El-Banna A.A., Pitt J.I., Leistner L. (1987). Production of mycotoxins by *Penicillium* species. Sys. Appl. Microbiol..

[21] Dorner J.W. (1983). Production of cyclopiazonic acid by *Aspergillus tamarii* Kita. Appl. Environ. Microbiol..

[22] Dorner J.W., Cole R.J., Lomax L.G., Gosser H.S., Diener U.L. (1983). Cyclopiazonic acid production by *Aspergillus flavus* and its effects on broiler chickens. Appl. Environ. Microbiol..

[23] Dorner J.W., Cole R.J., Diener U.L. (1984). The relationship of *Aspergillus flavus* and *Aspergillus parasiticus* with reference to production of aflatoxins and cyclopiazonic acid. Mycopathologia.

[24] Vinokurova N.G., Ivanushkina N.E., Khmel'nitskaia, Arinbasarov M.U. (2007). Synthesis of alpha-cyclopiazonic acid by fungi of the genus *Aspergillus*. Prikl. Biokhim. Mikrobiol..

[25] Ohmomo S., Sugita M., Matazo A. (1973). Isolation of cyclopiazonic acid, cyclopiazonic acid imine and bissecodehydrocyclopiazonic acid from the cultures of *Aspergillus versicolor* (Vuill.) Tiraboschi. J. Agr. Chem. Soc. Jpn..

[26] Domsch K.H., Gams W., Anderson T.-H. (1980). Compendium of Soil Fungi.

[27] Vaamonde G., Patriarca A., Fernandez Pinto V., Comerio R., Degrossi C. (2003). Variability of aflatoxin and cyclopiazonic acid production by *Aspergillus* section *Flavi* from different substrates in Argentina. Int. J. Food Microbiol..

[28] Pildain M.B., Vaamonde G., Cabral D. (2004). Analysis of population structure of *Aspergillus flavus* from peanut based on vegetative compatibility, geographic origin, mycotoxin and sclerotia production. Int. J. Food Microbiol..

[29] Novas M.V., Cabral D. (2002). Association of mycotoxin and sclerotia production with compatibility groups in *Aspergillus flavus* from peanut in Argentina. Plant Dis..

[30] Barros G., Torres A., Chulze S. (2005). *Aspergillus flavus* population isolated from soil of Argentina's peanut growing region. Sclerotia production and toxigenic profile. J. Sci. Food Agric..

[31] Romero S.M., Comerio R.M., Larumbe G., Ritieni A., Vaamonde G., Fernandez Pinto V. (2005). Toxigenic fungi isolated from dried vine fruits in Argentina. Int. J. Food Microbiol..

[32] Cvetnic Z., Pepeljnjak S. (1998). Production of cyclopiazonic acid by aflatoxigenic and non-aflatoxigenic strains of *Aspergillus flavus*. Nahrung.

[33] Richard J.L., Bhatnagar D., Peterson S., Sandor G. (1992). Assessment of aflatoxin and cyclopiazonic acid production by *Aspergillus flavus* isolates from Hungary. Mycopathologia.

[34] Bamba R., Sumbali G. (2005). Co-occurrence of aflatoxin B_1_ and cyclopiazonic acid in sour lime (*Citrus aurantifolia* Swingle) during post-harvest pathogenesis by *Aspergillus flavus*. Mycopathologia.

[35] Razzaghi-Abyaneh M., Shams-Ghahfarokhi M., Allameh A., Kazeroon-Shiri A., Ranjbar-Bahadori S., Mirzahoseini H., Rezaee M.B. (2006). A survey on distribution of *Aspergillus* section *Flavi* in corn field soils in Iran: Population patterns based on aflatoxins, cyclopiazonic acid and sclerotia production. Mycopathologia.

[36] Lisker N., Michaeli R., Frank Z.R. (1993). Mycotoxigenic potential of *Aspergillus flavus* strains isolated from groundnuts growing in Israel. Mycopathologia.

[37] Giorni P., Magan N., Pietri A., Bertuzzi T., Battilani P. (2007). Studies on *Aspergillus* section *Flavi* isolated from maize in northern Italy. Int. J. Food Microbiol..

[38] Rodrigues P., Venancio A., Kozakiewicz Z., Lima N. (2009). A polyphasic approach to the identification of aflatoxigenic and non-aflatoxigenic strains of *Aspergillus* section *Flavi* isolated from Portuguese almonds. Int. J. Food Microbiol..

[39] Blaney B.J., Kelly M.A., Tyler A.L., Connole M.D. (1989). Aflatoxin and cyclopiazonic acid production by Queensland isolates of *Aspergillus flavus* and *Aspergillus parasiticus*. Aust. J. Agric. Res..

[40] Sanchez-Hervas M., Gil J.V., Bisbal F., Ramon D., Martinez-Culebras P.V. (2008). Mycobiota and mycotoxin producing fungi from cocoa beans. Int. J. Food Microbiol..

[41] Lee Y.J., Hagler W.M.J. (1991). Aflatoxin and cyclopiazonic acid production by *Aspergillus flavus* isolated from contaminated maize. J. Food Sci..

[42] Horn B.W., Dorner J.W. (1999). Regional differences in production of aflatoxin B_1_ and cyclopiazonic acid by soil isolates of *Aspergillus flavus* along a transect within the United States. Appl. Environ. Microbiol..

[43] Gallagher R.T., Richard J.L., Stahr H.M., Cole R.J. (1978). Cyclopiazonic acid production by aflatoxigenic and non-aflatoxigenic strains of *Aspergillus flavus*. Mycopathologia.

[44] Urano T., Trucksess M.W., Beaver R.W., Wilson D.M., Dorner J.W., Dowell F.E. (1992). Co-occurrence of cyclopiazonic acid and aflatoxins in corn and peanuts. J. Off. Anal. Chem. Int..

[45] Widiastuti R., Maryam R., Blaney B.J., Salfina, Stoltz D.R. (1988). Cyclopiazonic acid in combination with aflatoxins, zearalenone and ochratoxin A in Indonesian corn. Mycopathologia.

[46] Mphande F.A., Siame B.A., Taylor J.E. (2004). Fungi, aflatoxins and cyclopiazonic acid associated with peanut retailing in Botswana. J. Food Prot..

[47] Hayashi Y., Yoshizawa T. (2005). Survey of cyclopiazonic acid contamination in corn from China and Southeast Asian countries. Mycotoxins.

[48] Lansden J.A., Davidson J.I. (1983). Occurrence of cyclopiazonic acid in peanuts. Appl. Environ. Microbiol..

[49] Castro M., Soares L., Furlani R. (1995). Mycoflora, aflatoxigenic species and mycotoxins in freshly harvested corn (*Zea mays* L.): A preliminary study. Rev. Microbiol..

[50] Adebajo L.O., Idowu A.A., Adesanya O.O. (1994). Mycoflora, and mycotoxins production in Nigerian corn and corn-based snacks. Mycopathologia.

[51] Goncalez E., Nogueira J.H., Fonseca H., Felicio J.D., Pino F.A., Correa B. (2008). Mycobiota and mycotoxins in Brazilian peanut kernels from sowing to harvest. Int. J. Food Microbiol..

[52] Bhattacharya K., Raha S. (2002). Deteriorative changes of maize, groundnut and soybean seeds by fungi in storage. Mycopathologia.

[53] Khosravi A.R., Mansouri M., Bahonar A.R., Shokri H. (2007). Mycoflora of maize harvested from Iran and imported maize. Pak. J. Biol. Sci..

[54] Ito Y., Peterson S.W., Wicklo D.T., Goto T. (2001). *Aspergillus pseudotamarii*, a new aflatoxin producing species in *Aspergillus* section *Flavi*. Mycological Res..

[55] Peterson S.W., Ito Y., Horn B.W., Goto T. (2001). *Aspergillus bombycis*, a new aflatoxigenic species and genetic variation in its sibling species, *A. nomius*. Mycologia.

[56] Hesseltine C.W., Shotwell O.D., Smith M., Ellis J.J., Vandergraft E.E., Shannon G.M., Herzberg M. (1970). Production of various aflatoxins bystrains of the *Aspergillus flavus* series. Toxic Microorganisms: Mycotoxin, Botulism.

[57] Cotty P.J. (1989). Virulence and cultural characteristics of two *Aspergillus flavus* strains pathogenic on cotton. Phytopathology.

[58] Geiser D.M., Dorner J.W., Horn B.W., Taylor J.W. (2000). The phylogenetics of mycotoxin and sclerotium production in *Aspergillus flavus* and *Aspergillus oryzae*. Fungal Genet. Biol..

[59] Saito M., Tsuruta O. (1993). A new variety of *Aspergillus flavus* from tropical soil in Thailand and its aflatoxin productivity. Proc. Jpn. Assoc. Mycotoxicol..

[60] Ehrlich K.C., Kobbeman K., Montalbano B.G., Cotty P.J. (2007). Aflatoxin-producing *Aspergillus* species from Thailand. Int. J. Food Microbiol..

[61] Cotty P.J., Cardwell K.F. (1999). Divergence of West African and North American communities of *Aspergillus* section *Flavi*. Appl. Environ. Microbiol..

[62] Pildain M.B., Frisvad J.C., Vaamonde G., Cabral D., Varga J., Samson R.A. (2008). Two novel aflatoxin-producing *Aspergillus* species from Argentinean peanuts. Int. J. Syst. Evol. Microbiol..

[63] Luk K.C., Kobbe B., Townsend J.M. (1977). Production of cyclopiazonic acid by *Aspergillus flavus* Link. Appl. Environ. Microbiol..

[64] Riley R.T., Goeger D.E., Yoo H., Showker J.L. (1992). Comparison of three tetramic acids and their ability to alter membrane function in cultured skeletal muscle cells and sarcoplasmic reticulum vesicles. Toxicol. Appl. Pharmacol..

[65] Goeger D.E., Riley R.T., Dorner J.W., Cole R.J. (1988). Cyclopiazonic acid inhibition of the Ca^2+^-transport ATPase in rat skeletal muscle sarcoplasmic reticulum vesicles. Biochem. Pharmacol..

[66] Moncoq K., Trieber C.A., Young H.S. (2007). The molecular basis for cyclopiazonic acid inhibition of the sarcoplasmic reticulum calcium pump. J. Biol. Chem..

[67] Purchase I.F. (1971). The acute toxicity of the mycotoxin cyclopiazonic acid to rats. Toxicol. Appl. Pharmacol..

[68] Nishie K., Cole R.J., Dorner J.W. (1987). Toxic effects of cyclopiazonic acid in the early phase of pregnancy in mice. Res. Commun. Chem. Pathol. Pharmacol..

[69] Nuehring L.P., Rowland G.N., Harrison L.R., Cole R.J., Dorner J.W. (1985). Cyclopiazonic acid mycotoxicosis in the dog. Am. J. Vet. Res..

[70] Lomax L.G., Cole R.J., Dorner J.W. (1984). The toxicity of cyclopiazonic acid in weaned pigs. Vet. Pathol..

[71] Smith E.E., Kubena L.F., Braithwaite C.E., Harvey R.B., Phillips T.D., Reine A.H. (1992). Toxicological evaluation of aflatoxin and cyclopiazonic acid in broiler chickens. Poult. Sci..

[72] Keblys M., Bernhoft A., Hofer C.C., Morrison E., Larsen H.J., Flaoyen A. (2004). The effects of the *Penicillium* mycotoxins citrinin, cyclopiazonic acid, ochratoxin A, patulin, penicillic acid, and roquefortine C on *in vitro* proliferation of porcine lymphocytes. Mycopathologia.

[73] Bernhoft A., Keblys M., Morrison E., Larsen H.J., Flaoyen A. (2004). Combined effects of selected *Penicillium* mycotoxins on *in vitro* proliferation of porcine lymphocytes. Mycopathologia.

[74] Gentles A., Smith E.E., Kubena L.F., Duffus E., Johnson P., Thompson J., Harvey R.B., Edrington T.S. (1999). Toxicological evaluations of cyclopiazonic acid and ochratoxin A in broilers. Poult. Sci..

[75] Kubena L.F., Smith E.E., Gentles A., Harvey R.B., Edrington T.S., Phillips T.D., Rottinghaus G.E. (1994). Individual and combined toxicity of T-2 toxin and cyclopiazonic acid in broiler chicks. Poult. Sci..

[76] Venkatesh P.K., Vairamuthu S., Balachandran C., Manohar B.M., Raj G.D. (2005). Induction of apoptosis by fungal culture materials containing cyclopiazonic acid and T-2 toxin in primary lymphoid organs of broiler chickens. Mycopathologia.

[77] Kamalavenkatesh P., Vairamuthu S., Balachandran C., Manohar B.M., Raj G.D. (2005). Immunopathological effect of the mycotoxins cyclopiazonic acid and T-2 toxin on broiler chicken. Mycopathologia.

[78] Antony M., Shukla Y., Janardhanan K.K. (2003). Potential risk of acute hepatotoxicity of kodo poisoning due to exposure to cyclopiazonic acid. J. Ethnopharmacol..

[79] Bradburn N., Coker R.D., Blunden G. (1994). The aetiology of turkey 'X' disease. Phytochemistry.

[80] Spensley P.C. (1963). Aflatoxin, the active principle in turkey 'X' disease. Endeavour.

[81] Cole R.J. (1986). Etiology of turkey "X" disease in retrospect: A case for the involvement of cyclopiazonic acid. Mycotoxin Res..

[82] Rao L.B., Husain A. (1985). Presence of cyclopiazonic acid in kodo millet (*Paspalum scrobiculatum*) causing 'kodua poisoning' in man and its production by associated fungi. Mycopathologia.

[83] Holzapfel C.W., Wilkins D.C. (1971). On the biosynthesis of cyclopiazonic acid. Phytochemistry.

[84] McGrath R.M., Steyn P.S., Ferreira N.P., Neethling D.C. (1976). Biosynthesis of cyclopiazonic acids in *Penicillium cyclopium*: The Isolation of dimethylallylpyrophosphate: Cyclo-acetoacetyltryptophanyl dimethylallyltransferase. Biorg. Chem..

[85] Agurell S., Lindgren J.E. (1968). Natural occurrence of 4-dimethylallyltryptophan-an ergot alkloid precursor. Tetrahedron Lett..

[86] Gebler J.C., Poulter C.D. (1992). Purification and characterization of dimethylallyl tryptophan synthase from *Clavicepsa purpurea*. Arch. Biochem. Biophys..

[87] Plieninger H., Immel H., Volkl A. (1967). Synthesis and incorporation of a 14C-and 3H-labeled 4-dimethylallyl-tryptophan and a 14C-labeled 4-dimethylallyl-tryptamine as well as incorporation of a 14C-labelled dimethylallyl-pyrophosphate. Justus Liebigs Ann. Chem..

[88] Robbers J.E., Floss H.G. (1968). Biosynthesis of ergot alkaloids: Formation of 4-dimethylallyltryptophan by the ergot fungus. Arch. Biochem. Biophys..

[89] McGrath R.M., Steyn P.S., Ferreira N.P. (1973). α-Acetyl-γ-(β-indolyl)methyltetramic acid. A biosynthetic intermediate of cyclopiazonic acid and of bis-secodehydrocyclopiazonic acid. J. Chem. Soc.,Chem. Commun..

[90] Heinstein P.F., Lee S.I., Floss H.G. (1971). Isolation of dimethylallylpyrophosphate: Tryptophan dimethylallyl transferase from the ergot fungus (*Claviceps* spec.). Biochem. Biophys. Res. Commun..

[91] McGrath R.M., Steyn P.S., Nourse P.N., Neethling D.C., Ferreira N.P. (1977). The diversion of dimethylallyl pyrophosphate from polyisoprenoid to cyclopiazonic acid in *Penicillium cyclopium* Westling. Biorg. Chem..

[92] Steffan N., Grundmann A., Yin W.B., Kremer A., Li S.M. (2009). Indole prenyltransferases from fungi: A new enzyme group with high potential for the production of prenylated indole derivatives. Curr. Med. Chem..

[93] Schabort J.C., Wilkens D.C., Holzapfel C.W., Potgieter D.J., Neitz A.W. (1971). β-cyclopiazonate oxidocyclase from *Penicillium cyclopium*. I. Assay methods,isolation and purification. Biochim. Biophys. Acta.

[94] Steenkamp D.J., Schabort J.C., Holzapfel C.W., Ferreira N.P. (1974). The role of essential histidines in the mechanism of catalysis of the flavoenzyme, β-cyclopiazonate oxidocyclase. Biochim. Biophys. Acta.

[95] Steenkamp D.J., Schabort J.C., Ferreira N.P. (1973). β-cyclopiazonate oxidocyclase from *Penicillium cyclopium*. 3. Preliminary studies on the mechanism of action. Biochim. Biophys. Acta.

[96] Wilding E.I., Brown J.R., Bryant A.P., Chalker A.F., Holmes D.J., Ingraham K.A., Iordanescu S., So C.Y., Rosenberg M., Gwynn M.N. (2000). Identification, evolution, and essentiality of the mevalonate pathway for isopentenyl diphosphate biosynthesis in gram-positive cocci. J. Bacteriol..

[97] Andreassi J.L., Vetting M.W., Bilder P.W., Roderick S.L., Leyh T.S. (2009). Structure of the ternary complex of phosphomevalonate kinase: The enzyme and its family. Biochemistry.

[98] Durbecq V., Sainz G., Oudjama Y., Clantin B., Bompard-Gilles C., Tricot C., Caillet J., Stalon V., Droogmans L., Villeret V. (2001). Crystal structure of isopentenyl diphosphate:dimethylallyl diphosphate isomerase. EMBO J..

[99] Machida M., Yamada O., Gomi K. (2008). Genomics of *Aspergillus oryzae*: Learning from the history of koji mold and exploration of its future. DNA Res..

[100] Payne G.A., Nierman W.C., Wortman J.R., Pritchard B.L., Brown D., Dean R.A., Bhatnagar D., Cleveland T.E., Machida M., Yu J. (2006). Whole genome comparison of *Aspergillus flavus* and *A. oryzae*. Med. Mycol..

[101] Chang P.-K., Horn B.W., Dorner J.W. (2005). Sequence breakpoints in the aflatoxin biosynthesis gene cluster and flanking regions in nonaflatoxigenic *Aspergillus flavus* isolates. Fungal Genet. Biol..

[102] Christensen B.E., Mollgaard H., Kaasgaard S., Lehmbeck J. (2002). Methods for producing polypeptides in *Aspergillus* mutant cells.

[103] Christensen B.E., Mollgaard H., Kaasgaard S., Lehmbeck J. (2007). Methods for producing polypeptides in *Aspergillus* mutant cells.

[104] Ehrlich K.C., Montalbano B.G., Cary J.W. (1999). Binding of the C6-zinc cluster protein, AFLR, to the promoters of aflatoxin pathway biosynthesis genes in *Aspergillus parasiticus*. Gene.

[105] Gulick A.M. (2009). Conformational dynamics in the acyl-CoA synthetases, adenylation domains of non-ribosomal peptide synthetases, and firefly luciferase. ACS Chem. Biol..

[106] Rausch C., Weber T., Kohlbacher O., Wohlleben W., Huson D.H. (2005). Specificity prediction of adenylation domains in nonribosomal peptide synthetases (NRPS) using transductive support vector machines (TSVMs). Nucleic Acids Res..

[107] Stachelhaus T., Marahiel M.A. (1995). Modular structure of peptide synthetases revealed by dissection of the multifunctional enzyme GrsA. J. Biol. Chem..

[108] Finking R., Marahiel M.A. (2004). Biosynthesis of nonribosomal peptides1. Annu. Rev. Microbiol..

[109] Fischbach M.A., Walsh C.T. (2006). Assembly-line enzymology for polyketide and nonribosomal peptide antibiotics: Logic, machinery, and mechanisms. Chem. Rev..

[110] Lin S., Van Lanen S.G., Shen B. (2009). A free-standing condensation enzyme catalyzing ester bond formation in C-1027 biosynthesis. Proc. Natl. Acad. Sci. U S A.

[111] Liu X., Walsh C.T. (2009). Cyclopiazonic acid biosynthesis in *Aspergillus* sp.: Characterization of a reductase-like R* domain in cyclopiazonate synthetase that forms and releases cyclo-acetoacetyl-L-tryptophan. Biochemistry.

[112] Metzger U., Schall C., Zocher G., Unsold I., Stec E., Li S.M., Heide L., Stehle T. (2009). The structure of dimethylallyl tryptophan synthase reveals a common architecture of aromatic prenyltransferases in fungi and bacteria. Proc. Natl. Acad. Sci. USA.

[113] Kenney W.C., Edmondson D.E., Singer T.P., Steenkamp D.J., Schabort J.C. (1974). The covalently bound flavin prosthetic group of β-cyclopiazonate oxidocyclase. FEBS Lett..

[114] Kenney W.C., Edmondson D.E., Singer T.P., Steenkamp D.J., Schabort J.C. (1976). Identification and properties of the covalently bound flavin of β-cyclopiazonate oxidocyclase. Biochemistry.

[115] Edmondson D.E., Binda C., Mattevi A. (2007). Structural insights into the mechanism of amine oxidation by monoamine oxidases A and B. Arch. Biochem. Biophys..

[116] Edmondson D.E., Binda C., Wang J., Upadhyay A.K., Mattevi A. (2009). Molecular and mechanistic properties of the membrane-bound mitochondrial monoamine oxidases. Biochemistry.

[117] Fitzpatrick P.F. (2009). Oxidation of amines by flavoproteins. Arch. Biochem. Biophys..

[118] Winkler A., Lyskowski A., Riedl S., Puhl M., Kutchan T.M., Macheroux P., Gruber K. (2008). A concerted mechanism for berberine bridge enzyme. Nat. Chem. Biol..

[119] Winkler A., Motz K., Riedl S., Puhl M., Macheroux P., Gruber K. (2009). Structural and mechanistic studies reveal the functional role of bicovalent flavinylation in berberine bridge enzyme. J. Biol. Chem..

[120] Winkler A., Puhl M., Weber H., Kutchan T.M., Gruber K., Macheroux P. (2009). Berberine bridge enzyme catalyzes the six electron oxidation of (*S*)-reticuline to dehydroscoulerine. Phytochemistry.

[121] Ehrlich K.C., Yu J., Cotty P.J. (2005). Aflatoxin biosynthesis gene clusters and flanking regions. J. Appl. Microbiol..

[122] Tokuoka M., Takahashi T., Seshime Y., Fujii I., Kitamoto K., Koyama Y. (2008). Identification of the biosynthetic pathway of cyclopiazonic acid in Aspergillus oryzae.

[123] Chang P.K., Ehrlich K.C., Hua S.S. (2006). Cladal relatedness among *Aspergillus oryzae* isolates and *Aspergillus flavus* S and L morphotype isolates. Int. J. Food Microbiol..

[124] Kusumoto K., Nogata Y., Ohta H. (2000). Directed deletions in the aflatoxin biosynthesis gene homolog cluster of *Aspergillus oryzae*. Curr. Genet..

[125] Tominaga M., Lee Y.H., Hayashi R., Suzuki Y., Yamada O., Sakamoto K., Gotoh K., Akita O. (2006). Molecular analysis of an inactive aflatoxin biosynthesis gene cluster in *Aspergillus oryzae* RIB strains. Appl. Environ. Microbiol..

[126] Riley R.T., Goeger D.E., Deepak Bhatnagar D., Lillehoj E.B., Arora D.K. (1992). Cyclopiazonic acid: Speculation on its function on fungi. Handbook of Applied Mycology: Mycotoxins in Ecological Systems.

